# SG-APSIC1066: Costs and surgical-site infection outcomes using ChloraPrep versus aqueous povidone iodine after colorectal surgeries in Australian public hospitals

**DOI:** 10.1017/ash.2023.89

**Published:** 2023-03-16

**Authors:** Kristin Hui Xian Tan, Yan Ma

**Affiliations:** Becton Dickinson, Singapore; Becton Dickinson, Singapore

## Abstract

**Objectives:** In Australia, the prevalence of SSI is 3.6%, with a particularly high burden in colorectal procedures of 8.7%. ChloraPrep (2% chlorhexidine gluconate (CHG)–70% isopropyl alcohol formulation) is a preoperative sterile alcoholic CHG solution prefilled in a ready-to-use applicator. We compared the costs and SSI outcomes of adopting ChloraPrep versus a bulk aqueous povidone iodine (PVI) solution for colorectal procedures in a public hospital setting. **Methods:** We used a budget-impact tool to evaluate the clinical and economic impacts of skin preparation methods. The PVI SSI rate was assumed to be the baseline rate observed in Australia (8.7%). The ChloraPrep SSI rate was derived by applying the demonstrated ChloraPrep SSI reduction versus PVI (41%) to the PVI SSI rate. The cost of ChloraPrep was AU$8 (US $5.50) and the cost of PVI solution was AU$3 (US $2). The PVI equipment cost AU$2.13 (US $1.47). Additional average length of stay was 9.4 days, and the daily average cost was AU$2,347 (US $1,618). The average skin preparation time was 3.5 minutes using ChloraPrep and 8.5 minutes using PVI. The hospital-acquired complication (HAC) penalty for SSI was calculated using the national efficient price (AU $5,797 or US $3,996), national weighted activity unit (4.6261), and adjustment rate for patient complexity levels (high, 4.8%; moderate, 5.9%; and low, 7.9%). **Results:** The model estimated SSI rates were 5.1% using ChloraPrep and 8.7% for PVI. For every 1,000 patients, skin preparation cost was estimated to be AU$8,100 (US $5.583) using ChloraPrep and AU$5,200 (US $3.585) using PVI. SSI treatment cost was estimated to be AU$449,900 (US $310,127) for ChloraPrep and AU$762,500 (US $525,610) for PVI. In addition, 330 bed days could be avoided and at least 80 operating room hours could be saved with 35 SSIs avoided. With 35 SSIs avoided, a potential reduction of AU$26,500 (US $18,267) in HAC penalty could be expected. This intervention could yield an overall cost savings of AU$336,300 (US $231,820). **Conclusions:** Using ChloraPrep for skin preparation prior to colorectal procedures could result in lower SSI rates and cost savings from treating fewer SSIs. Operational efficiency might also be improved.

